# Economic Impact of Malignant Catarrhal Fever on Cattle Production in Lephalale Municipality, Limpopo Province, South Africa

**DOI:** 10.3390/vetsci13030305

**Published:** 2026-03-23

**Authors:** Walter Shiba, Itumeleng Matle, Siphe Zantsi, Emmanuel Seakamela

**Affiliations:** 1 Economic Analysis Unit, Agricultural Research Council, 1134 Park Street, Pretoria 0028, South Africa; zantsis@arc.agric.za; 2 Department of Agriculture and Animal Health, Science Campus, University of South Africa, Roodepoort 1709, South Africa; 3 Department of Agriculture and Animal Health, University of South Africa, 28 Pioneer Ave, Florida Park, Roodepoort 1709, South Africa; 4 Department of Agricultural Science, Nelson Mandela University, Gqeberha 6031, South Africa; 5 Bacteriology Section, Agricultural Research Council: Onderstepoort Veterinary Research, Onderstepoort 0110, South Africa; seakamelae@arc.agric.za

**Keywords:** livestock economics, rural livelihoods, malignant catarrhal fever, wildlife–livestock interface, Africa

## Abstract

Malignant catarrhal fever (MCF) is a highly lethal viral disease of cattle that poses a persistent threat to livestock production in wildlife–livestock interface areas of Southern Africa. Despite its recognized clinical severity, the economic burden of the disease remains poorly quantified in South African production systems. This study assessed the long-term economic impact of malignant catarrhal fever on cattle production in Lephalale Municipality, Limpopo Province, South Africa, using a retrospective analysis covering the period from 2001 to 2021. The study combined confirmed case records, estimated mortalities, and region-specific production parameters to quantify both direct and indirect economic losses. Direct losses included mortality-related financial costs and the expenditure on treatment, while indirect losses encompassed reduced productivity, diminished milk yields, and associated declines in overall herd performance. The results show that MCF imposed substantial financial burdens on cattle producers, with mortality contributing to more than ninety percent of total losses. The total economic losses over the study period were substantial, amounting to approximately R 1.55 million, driven primarily by high mortality-related costs. Annual losses displayed considerable variability, reflecting the sporadic nature of the outbreaks and the fluctuations in the wildlife–livestock interactions. The spatial analysis revealed that most cases occur in wards situated adjacent to wildlife conservation areas, where cattle are exposed to virus-carrying wildebeest populations. Seasonal patterns indicated a higher disease occurrence during the spring and winter, aligning with established transmission dynamics. Although a gradual decline in both cases and associated losses was observed over the study period, episodic outbreaks continued to exert significant economic shocks on affected farming households. The findings reaffirm MCF as a persistent and high-impact constraint to cattle production in interface zones and highlight the need for improved surveillance, integrated land-use planning, and sustained investment in targeted disease control measures.

## Simple Summary

Malignant catarrhal fever (MCF) is a deadly cattle disease that often occurs where livestock and wildlife share grazing areas. In South Africa, farmers living near conservation zones are especially vulnerable, yet the financial impact of the disease is not well understood. This study examined 21 years of data (2001–2021) from Lephalale Municipality to estimate how much money farmers lose when MCF outbreaks occur. We calculated direct losses, such as cattle deaths and treatment costs, as well as indirect losses from reduced productivity and lower milk production. Our results show that MCF caused major economic damage, mainly because most infected cattle die. The losses were the highest in the areas closest to wildlife and during spring and winter. Although cases have declined over time, occasional outbreaks still create severe financial shocks for farmers. The findings highlight the need for stronger disease surveillance, better land-use planning, and long-term strategies to protect cattle in wildlife–livestock interface areas.

## 1. Introduction

Livestock production remains a cornerstone of rural livelihoods across sub–Saharan Africa, providing income, food security, employment, and a critical store of wealth for millions of households. In South Africa, cattle are particularly important in communal and smallholder systems, as they contribute to household nutrition, draught power, cultural practices, and social capital, while also functioning as a buffer against economic shocks such as drought, unemployment, and market volatility [1,2]. Livestock assets are therefore not only productive inputs but also central to livelihood resilience, with losses having long-lasting implications on poverty dynamics and rural development trajectories.

However, livestock-based livelihoods in South Africa face persistent constraints from animal diseases that are increasingly shaped by environmental change, land use transformation, and the expansion of wildlife–livestock interfaces. The coexistence of livestock production and wildlife conservation has intensified the exposure to infectious diseases that are maintained in wildlife reservoirs, particularly in regions where communal grazing areas overlap spatially with protected areas and private game farms [3,4]. These interface zones are especially prominent in the Limpopo Province, where cattle production systems operate alongside wildlife conservation and tourism activities, creating complex trade-offs between economic development, conservation objectives, and animal health risks.

Lephalale Municipality is characterized by extensive and predominantly communal livestock farming systems, where cattle production forms a central component of household livelihoods and local economies. The area comprises open grazing rangelands, mixed veld types, and a climate marked by hot summers and dry winters, which are all conditions that shape forage availability and seasonal herd movements. Cattle are kept primarily for meat production, social functions, and as financial assets, with a limited adoption of intensive production technologies. Importantly, Lephalale shares long ecological boundaries with private game reserves and conservation zones containing free-ranging blue wildebeest, creating a typical wildlife–livestock interface. In these unfenced or partially fenced landscapes, cattle frequently graze in proximity to wildebeest, increasing the risk of pathogen transmission. The communal nature of grazing, combined with constrained veterinary service access, low barriers to wildlife contact, and a limited disease-prevention infrastructure, makes the region particularly vulnerable to high-impact diseases such as malignant catarrhal fever.

MCF is caused by a group of gammaherpesviruses collectively referred to as malignant catarrhal fever viruses (MCFV), of which Alcelaphine herpesvirus-1 (AlHV-1) and Ovine herpesvirus-2 (OvHV-2) are the most clinically important. AlHV-1 is maintained asymptomatically in wildebeest, particularly during the calving season when viral shedding is at its highest, while OvHV-2 circulates in domestic sheep populations. Cattle are dead-end hosts in which infection typically results in a peracute or acute fatal disease, with case-fatality rates frequently exceeding 80%. The transmission to cattle occurs primarily through close contact with shedding wildebeest calves or infected sheep, although infected cattle do not transmit the virus further. The laboratory detection of MCFV through the PCR amplification of viral DNA is fundamental for confirming clinical diagnoses, distinguishing the disease from other causes of acute febrile illness, and guiding outbreak investigation. As no effective vaccine or curative treatment exists, early detection of MCFV and accurate identification of the transmission source remain essential components of disease prevention, risk communication, and herd-level management [5,6]. In Southern Africa, wildebeest-associated malignant catarrhal fever represents the dominant transmission pathway, with seasonal viral shedding during calving periods substantially increasing infection risk for cattle that are grazing in proximity to wildlife [7]. Although herd-level incidence is typically low, the near certainty of death following an infection means that even a small number of cases can generate disproportionate economic losses.

For smallholders and communal farmers, the loss of cattle due to malignant catarrhal fever extends beyond immediate production losses to undermine asset accumulation, herd growth, and household resilience. Studies across Africa demonstrate that disease-induced livestock losses reduce herd size, constrain future income streams, and increase vulnerability to subsequent shocks, reinforcing long-term livelihood insecurity [5,8]. In systems where access to veterinary services, fencing, and alternative grazing land is limited, farmers have few viable options for disease prevention or risk mitigation.

Despite the recognized clinical severity of malignant catarrhal fever and its designation as a notifiable disease in South Africa, its economic burden remains poorly quantified. The existing research has focused primarily on epidemiology, pathology, and transmission dynamics, with limited attention given to systematic economic assessment. Where economic studies exist, they are often short-term, geographically limited, or based on pastoral systems outside South Africa, reducing their applicability to local policy contexts [5]. Moreover, national surveillance systems typically report disease occurrence without translating case data into economic loss metrics that capture mortality, productivity impacts, and long-term production setbacks. This lack of robust, context-specific economic evidence constrains disease prioritization, land use planning, and investment decisions related to wildlife–livestock coexistence.

The research gap is particularly evident in mixed farming systems such as those found in Lephalale Municipality, where longitudinal surveillance has confirmed the continued presence of MCF but has not quantified its contribution to cattle mortality or farm-level economic loss [9]. Without such evidence, the true burden of the disease remains underestimated, and policy responses risk being poorly targeted or insufficiently resourced.

Against this background, this paper makes three key contributions. First, it provides a long-term retrospective economic assessment of malignant catarrhal fever in a South African wildlife–livestock interface area, covering a twenty-one-year period. Second, it translates epidemiological data into disaggregated economic loss components, distinguishing between the direct losses from mortality and the treatment costs and the indirect losses from reduced productivity and milk output, following established animal health economics frameworks [10]. Third, it situates these losses within their spatial, seasonal, and temporal context, offering insights into how ecological exposure and institutional constraints shape economic vulnerability among cattle producers. In doing so, the study contributes the empirical evidence needed to inform targeted disease management strategies and policies aimed at safeguarding livestock-based rural livelihoods.

## 2. Theoretical Framework

This study is grounded in the economics of animal health and livestock production systems, drawing on disease impact theory, livelihood resilience frameworks, and the One Health perspective to explain how malignant catarrhal fever generates economic losses in the cattle production systems located at wildlife–livestock interfaces. The framework conceptualizes disease occurrence as an exogenous biological shock that interacts with farm-level characteristics, institutional constraints, and ecological contexts to produce direct and indirect economic losses over time.

At its core, animal health economics views livestock disease as a negative productivity shock that reduces the value of animal capital and disrupts production flows within farming systems [10,11]. In this perspective, cattle are treated as productive assets that generate streams of benefits, including meat, milk, draught power, breeding services, and social value. Disease events such as malignant catarrhal fever abruptly reduce or eliminate these benefit streams, either permanently through mortality or temporarily through morbidity and reduced productivity. Therefore, the magnitude of economic loss depends not only on biological severity but also on the economic role of the cattle within the production system.

In South African mixed farming systems, particularly those adjacent to wildlife conservation areas, disease risk is shaped by the ecological interactions between livestock, wildlife reservoirs, and seasonal environmental processes. Historical and contemporary studies demonstrate that transhumance, wildlife movements, and land use change intensify disease transmission at the wildlife–livestock interface, particularly for diseases with wildlife reservoirs such as malignant catarrhal fever [3,4]. Wildebeest-associated viral shedding during calving seasons creates predictable but difficult-to-manage exposure risks for cattle, especially where fencing, grazing alternatives, and land tenure arrangements are constrained.

The framework further draws on the livestock systems sustainability theory, which emphasizes that disease impacts must be understood within the broader constraints related to climate variability, production inputs, and adaptive capacity [11]. In extensive and smallholder systems, limited access to veterinary services, the absence of effective vaccines, and financial constraints restrict farmers’ ability to prevent or mitigate disease impacts. As a result, highly lethal diseases such as malignant catarrhal fever produce disproportionate economic losses even when the incidence is relatively low.

The empirical studies across Southern Africa show that livestock ownership and herd intensity are strongly influenced by socio-economic factors, risk exposure, and disease prevalence [8]. The losses from infectious diseases reduce herd size directly through mortality and indirectly through distress sales, reduced reproduction, and long-term disinvestment in livestock. These dynamics create feedback effects in which disease losses erode household resilience and increase vulnerability to future shocks, consistent with asset-based poverty trap theory [5,12].

Within this study, economic losses are conceptualized as occurring through two primary pathways. The direct losses arise from immediate expenditures and asset destruction associated with disease, notably cattle mortality and treatment-related costs. These losses are observable, quantifiable, and largely irreversible, particularly in systems where replacement of breeding stock is slow and costly. The indirect losses arise from reduced productivity among surviving animals, including declines in body condition, milk yield, and reproductive performance, as well as longer-term production setbacks that persist beyond the disease episode. The previous studies on livestock diseases such as heartwater, lumpy skin disease, Rift Valley fever, and malignant catarrhal fever consistently show that mortality dominates total economic loss, but indirect effects accumulate over time and exacerbate livelihood impacts [13,14,15].

The framework also incorporates the One Health approach, which recognizes that livestock disease dynamics are shaped by interactions between animal health, environmental processes, and human decision making. In the case of malignant catarrhal fever, wildlife conservation policies, land use planning, and livestock management practices jointly determine exposure risk and feasible control strategies. The absence of an effective vaccine shifts the burden of disease management toward avoidance strategies such as spatial separation, altered grazing patterns, and seasonal movement restrictions, all of which impose implicit economic costs on farmers [5].

The theoretical framework positions malignant catarrhal fever as a high-impact, low-frequency shock within livestock production systems. Economic losses emerge from the interaction between biological lethality, exposure to the wildlife–livestock interface, farm-level vulnerability, and a limited adaptive capacity. This study operationalizes the framework by quantifying direct and indirect losses over time, thereby translating epidemiological outcomes into economic metrics that are relevant for policy design, disease prioritization, and land use decision-making.

## 3. Methodology

### 3.1. Study Design and Data Sources

The study employed a retrospective economic impact assessment covering a 21-year period (2001–2021) [9]. The epidemiological data on laboratory-confirmed MCF cases and associated mortalities were obtained from the Lephalale Provincial Veterinary Laboratory diagnostic database and the official longitudinal surveillance records for Lephalale Municipality as reported by [9]. The economic parameters were derived from the published literature and expert consultations, including the average cattle value, the treatment costs, and the productivity loss rates [5,16,17]. Although the study period ended in 2021, more recent data (2022–2025) were not included because the ARC–Onderstepoort Veterinary Research (ARC-OVR) surveillance dataset used in this study had only undergone full validation up to the end of 2021 at the time of data extraction. The post-2021 records were still provisional, subject to routine laboratory auditing, case reconciliation, and data quality checks. Incorporating these unvalidated cases would have risked inconsistencies in diagnostic status, ward assignments, and mortality estimations. To ensure methodological consistency and data reliability, we restricted the analysis to the most recent fully verified surveillance window (2001–2021).

### 3.2. Analytical Framework

The economic losses were estimated using a component-based approach that distinguishes the direct losses (mortality and treatment costs) from the indirect losses (productivity and milk production losses) that are consistent with the international guidelines for animal health economics [10,18]. This framework enables a transparent attribution of costs and a sensitivity analysis of key parameters.

### 3.3. Parameter Assumptions

The case fatality rate (CFR) for MCF is estimated at 85%, which is consistent with the literature reporting mortality rates between 80% and 90% [5,17]. The average value of a cow is approximately R 6698.80, representing a conservative estimate for African cattle. The treatment costs per case are estimated at R 837.35, covering the veterinary consultation and supportive care. The productivity losses during illness are projected at 18%, reflecting the decline in body condition, while milk losses account for 8% of annual income during the disease season [5]. Given that the annual milk value per cow is approximately R 3349.40 based on regional market averages, these figures underscore the significant economic impact of the disease on smallholder farmers (Table 1), The full set of economic parameters, assumptions, and sensitivity ranges used in the loss calculations is provided in Supplementary Table S1.

### 3.4. Calculation Procedures

- The direct losses:
  - The mortality losses were calculated as the number of deaths multiplied by the average cattle value.
  - The treatment costs were computed as the number of positive cases multiplied by the treatment cost per case. The subtotal of direct losses = the mortality losses + the treatment costs.
- The indirect losses:
  - The productivity loss was estimated as the number of surviving cases multiplied by the cattle value and the productivity loss rate.
  - The milk loss was derived from the number of positive cases multiplied by the annual milk value and milk loss rate. The subtotal of indirect losses = productivity loss + milk loss.
- The total economic loss is the sum of the direct and indirect losses across the study period.

### 3.5. Trend and Correlation Analysis

The trend analysis is a time-series plot for the infection rate and the economic loss. Correlation: Pearson correlation coefficient:

$$r=\frac{\sum \left({IR}_{i}-I\bar{R})({EEL}_{i}-E\bar{E}L\right)}{\sqrt{\sum {(IR}_{i}}-I\bar{R})2\;\sum ({EEL}_{i}-E\bar{E}L)2}$$

### 3.6. Summary Metrics

The aggregate losses were normalized to compute the average annual loss, loss per positive case, and loss per death, along with percentage contributions of direct and indirect components. All monetary values were expressed in South African Rand (ZAR).

### 3.7. Quality Assurance

Internal consistency checks were performed to validate subtotal and total calculations. The discrepancies were flagged for correction prior to statistical analysis. The methodology aligns with the FAO and WOAH recommendations for livestock disease impact assessment [15,17].

The statistical analysis included descriptive measures and inferential tests to assess temporal trends and the variability in disease burden and associated losses. The descriptive statistics, such as mean, median, standard deviation, and coefficient of variation, were computed for the annual cases and economic losses to characterize the distributional properties. The temporal trends were evaluated using Pearson’s correlation to test for linear association between year and annual cases, complemented by Spearman’s rank correlation to confirm monotonic trends robust to non-normality. A Welch two-sample t-test compared the mean annual cases between early (2001–2010) and late (2011–2021) periods to account for unequal variances. The confidence intervals (95%) for average annual economic loss were estimated using t-distribution methods, and total loss intervals were derived by scaling mean bounds by the number of years. All analyses were performed in Microsoft Excel using standard functions and rank-based procedures, following the FAO and WOAH recommendations for livestock disease impact assessment [16,17].

## 4. Results

The annual case counts from 2001 to 2021 were analyzed using Pearson’s correlation to assess linear trends and Spearman’s rank correlation to confirm monotonic trends that are robust to non-normality. The comparison between the early (2001–2010) and the late (2011–2021) periods employed Welch’s two-sample *t*-test to accommodate unequal variances and sample sizes. Ninety-five percent confidence intervals for average annual economic loss were computed using *t*-intervals, and total loss intervals were derived by scaling the mean bounds by the number of years. The descriptive statistics, including mean, median, standard deviation, minimum–maximum range, and coefficient of variation, summarized the distributional properties. All analyses were conducted in Microsoft Excel using standard functions and rank-based procedures. The assumptions of independence and approximate normality were evaluated through rank-based confirmation and variance-robust testing.

### 4.1. Spatial Distribution of Positive Cases and Estimated Deaths by Ward (2001–2021)

**Figure 1:**
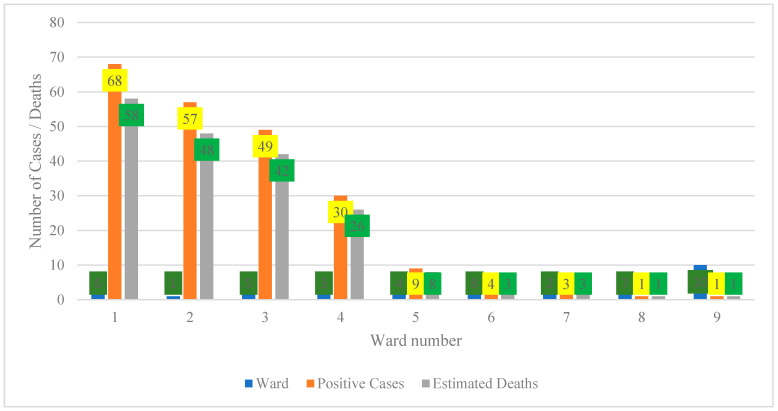
Spatial distribution of positive cases and estimated deaths by ward (2001–2021).

Figure 1 illustrates a pronounced concentration of positive cases and estimated deaths in wards 1 to 4, with ward 1 exhibiting the highest burden (68 positive cases and 58 estimated deaths), followed by ward 2 (57 cases and 48 deaths), ward 3 (49 cases and 42 deaths), and ward 4 (30 cases and 26 deaths). Collectively, these four wards account for approximately 84% of all positive cases and 92% of estimated deaths, indicating a substantial spatial clustering of disease burden. In contrast, wards 5 to 9 report markedly lower counts, ranging from 3 to 10 cases and one to nine estimated deaths, which may reflect a genuinely lower transmission or a potential underreporting that is attributable to limited surveillance and restricted access to diagnostic services [19]. in Figure 1 had zero confirmed cases or cases lacking reliable ward coordinates in the surveillance archive and were therefore excluded from the spatial figure to maintain data integrity.

An anomaly is evident in ward 1, where estimated deaths exceed recorded cases, resulting in an implied case fatality ratio greater than 100%. This pattern suggests that mortality figures are likely derived from population-based estimates or multi-year projections rather than direct outcomes of documented cases, necessitating clear annotation to prevent misinterpretation [20,21]. Comparable inconsistencies occur in other wards where deaths approach or surpass case counts, underscoring the importance of standardizing denominators and reporting mortality as rates per 100,000 population to ensure comparability [22].

From a policy perspective, the concentration of cases and deaths in wards 1–4 warrants prioritization of interventions such as targeted testing, case management, and risk communication within these areas [23,24]. Conversely, wards with low reported burden require enhanced community-level surveillance to ascertain whether low numbers reflect true incidence or under-detection. Overall, harmonization of case and mortality data and improvements in data quality are essential to strengthen the reliability of spatial analyses and support evidence-based resource allocation.

### 4.2. Seasonal Distribution of Disease Burden

**Figure 2:**
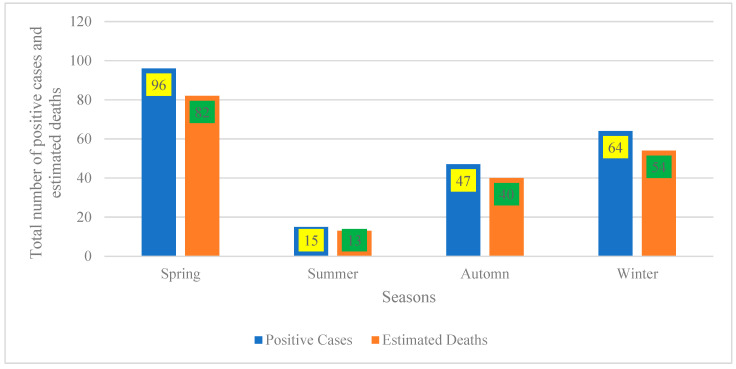
Seasonal distribution of positive cases and estimated deaths.

Figure 2 indicates that the seasonal distribution reveals two distinct peaks in disease burden: spring records the highest counts (96 positive cases; 82 estimated deaths), followed by a secondary peak in winter (64 cases; 54 deaths). Autumn exhibits moderate levels (47 cases; 40 deaths), while summer represents the trough (15 cases; 13 deaths). This pattern aligns with well-documented seasonality in infectious and zoonotic diseases, where incidence fluctuates across seasons due to interacting environmental drivers (e.g., temperature, rainfall, humidity), host and vector ecology (breeding and survival), and human or animal behaviors (occupational exposure, mobility, and contact networks) [25,26].

From a practical standpoint, these findings underscore the need to front-load surveillance and preparedness ahead of the spring and winter peaks. Recommended measures include intensified case finding, stockpiling of diagnostics and therapeutics, targeted risk communication, and, where applicable, pre-season vaccination or prophylaxis to mitigate peak-season transmission and mortality [21].

The proximity of estimated deaths to case counts across seasons (e.g., approximately 85% in spring and winter) suggests either a highly lethal syndrome or, more plausibly, that “estimated deaths” are derived from population-based models or multi-year rates rather than the same cohort of confirmed cases. To avoid misinterpretation, reports should standardize denominators and present season-specific rates per 100,000 population (with confidence intervals) rather than raw counts [21,22]. Finally, the pronounced summer trough warrants verification to distinguish true low incidence from under-detection (e.g., reduced testing access or reporting). If confirmed, this period could be leveraged for off-peak training, system maintenance, and data quality audits to ensure readiness for the subsequent spring peak [26].

### 4.3. Temporal Trends in Disease Burden

**Figure 3:**
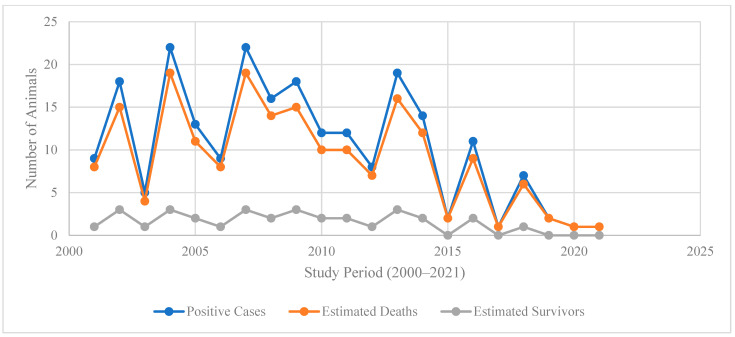
The annual trends in positive cases, estimated deaths, and survivors (2000–2021).

Trend analysis from 2000 to 2021 in Figure 3 illustrates a decline in positive cases and estimated deaths over time, with both indicators peaking between 2003 and 2007 (cases exceeding 20 per year and deaths closely following) before gradually decreasing after 2010. By 2020–2021, both cases and deaths approach near-zero levels, reflecting substantial progress in disease control and prevention efforts. This downward trajectory is consistent with global evidence that sustained interventions such as enhanced surveillance, vaccination programs, and community engagement reduce incidence and mortality of high-burden zoonotic and infectious diseases over time [21,27,28], A supplementary visualization of annual cases and estimated deaths, including smoothed trend lines, is provided in Supplementary Figure S1.

The close tracking of estimated deaths with positive cases throughout the period suggests a persistently high case fatality ratio, characteristic of certain neglected zoonoses, and underscores the importance of early detection and timely treatment [27,29]. However, the consistently low number of estimated survivors (generally fewer than four per year) highlights the limited success of treatment or the delayed access to care, reinforcing calls for integrated One Health strategies and improved health system responsiveness [27,28].

The sharp decline after 2015 may reflect intensified control measures, policy reforms, and increased resource allocation, as documented in recent regional and global reports on disease elimination programs [28]. Nevertheless, sporadic spikes in cases and deaths before 2015 indicate that outbreaks remained possible under conditions of surveillance gaps or ecological drivers such as climate variability and livestock movement [29,30]. Sustaining this downward trend will require a continued investment in preventive interventions, community awareness, and cross-sectoral collaboration to prevent resurgence [30].

### 4.4. Total Economic Losses in ZAR

**Figure 4:**
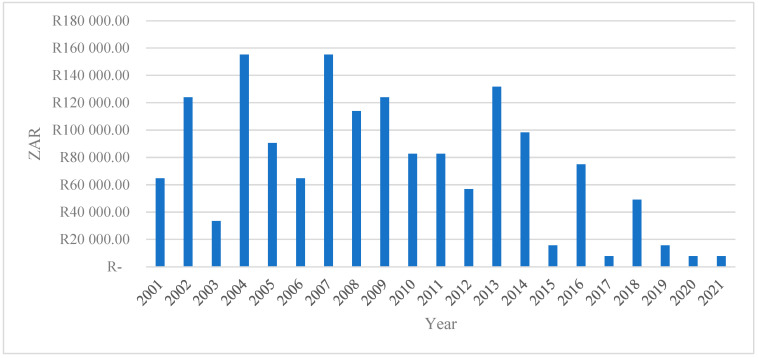
The total economic loss (ZAR).

Figure 4 depicts the total economic losses (measured in South African Rand) from 2001 to 2021. The series exhibits substantial interannual variability, indicating that economic losses are highly episodic rather than following a smooth or monotonic trend. This volatility suggests that losses are driven by discrete shocks or events rather than structural, gradual change.

The early 2000s (2001–2004) are characterized by moderate to high losses, with a pronounced spike around 2004, where total losses exceed ZAR 150,000. This peak may reflect severe economic or production shocks during this period, consistent with the literature showing that early transition and reform phases in developing economies are often associated with a heightened vulnerability to external and institutional disturbances [31,32].

Subsequently, the period between 2005 and 2010 shows persistently elevated losses, with several years exceeding ZAR 100,000. This pattern indicates a phase of sustained exposure to risk, potentially linked to cumulative shocks, weak resilience mechanisms, or a limited adaptive capacity. Empirical studies argue that repeated shocks can erode productive assets and amplify long-term economic losses, particularly in agriculture-dependent systems [33,34].

A notable decline in losses is observed after 2011, except for a sharp but isolated increase in 2013. This spike suggests a systemic disturbance occurring within an otherwise improving trend. Such isolated peaks are consistent with covariate shocks, such as droughts, market collapses, or policy disruptions that affect multiple economic agents simultaneously [35,36].

From 2015 onwards, total economic losses decline markedly and remain consistently low through to 2021. This sustained reduction may signal improvements in risk management, institutional response, technological adaptation, or structural resilience. The literature emphasizes that long-term declines in economic losses are often associated with improved early warning systems, diversification strategies, and stronger governance frameworks [37].

Overall, the figure highlights three key insights: (i) economic losses are non-linear and shock-driven, underscoring the importance of resilience-focused policy interventions; (ii) periods of repeated high losses suggest vulnerability traps, where actors struggle to recover fully between shocks; and (iii) the post-2015 decline indicates potential structural improvements that warrant further empirical investigation to identify the mechanisms driving reduced exposure and impact.

### 4.5. Aggregate Economic Losses by Category (2001–2021)

**Figure 5:**
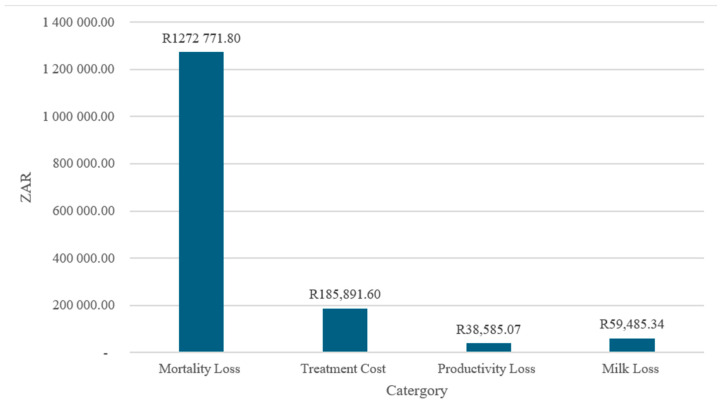
The aggregate losses by category (2001–2021)—ZAR.

Figure 5 illustrates the distribution of aggregate economic losses by category over the period 2001–2021. The composition of total losses is highly uneven, with mortality-related losses dominating the overall economic burden. The mortality losses amount to approximately ZAR 1.27 million, accounting for most of the total costs, while treatment costs (≈ZAR 186,000), milk losses (≈ZAR 59,000), and productivity losses (≈ZAR 39,000) contribute comparatively smaller shares.

The predominance of mortality losses suggests that economic impacts are driven primarily by irreversible asset losses rather than transitory production disruptions or disease management expenditures. This pattern aligns with evidence indicating that, in livestock and agri-food systems, mortality represents the most severe and least recoverable form of loss, particularly when outbreaks are detected late or preventive measures are insufficient [38]. High mortality costs also imply extended recovery periods, as herd rebuilding and genetic replacement require substantial time and capital investment.

The treatment costs constitute the second-largest loss category, reflecting recurrent expenditures on veterinary services, pharmaceuticals, and control measures. While these costs are non-negligible, their magnitude relative to mortality losses indicates that reactive disease management is substantially less costly than asset loss, reinforcing the economic rationale for preventive and early-intervention strategies [39].

Productivity and milk losses are comparatively modest, suggesting that affected production systems experience limited short-term output disruption relative to the scale of animal losses. However, the persistence of these losses over time implies cumulative impacts on farm income and household livelihoods, particularly for small-scale producers who rely on regular production flows for cash income and food security [40].

Overall, the loss composition underscores the central importance of prevention-focused policy and investment. Reducing mortality through improved surveillance, biosecurity, and rapid response mechanisms is likely to yield the largest economic returns, surpassing gains achievable through treatment or post-outbreak productivity recovery alone. These findings align with contemporary risk management frameworks that prioritize ex ante interventions over ex post compensation in agri-food systems [36,41].

### 4.6. Statistical Analysis Results

The temporal trend analysis reveals a statistically significant declining trend in annual cases over time. Both Pearson (r = −0.592, *p* < 0.01) and Spearman (ρ = −0.591, *p* < 0.01) correlation coefficients confirm a robust and monotonic decrease, indicating that the downward trend is not driven by short-term fluctuations but reflects a sustained structural change. The consistency between parametric and non-parametric tests strengthens confidence in the observed trend [42].

Period comparison analysis further corroborates this finding. Mean annual cases declined significantly from 14.40 cases/year (2001–2010) to 7.09 cases/year (2011–2021), with a statistically significant t-test result (t = 2.77, *p* = 0.012). This suggests that the later period experienced a meaningful reduction in incidence, likely reflecting improvements in prevention, management, or institutional response mechanisms rather than random variation. Similar declines have been associated with strengthened governance, early-warning systems, and targeted risk mitigation strategies in agri-food and public health systems [19,28].

The confidence interval estimates indicate that the total economic losses are substantial but precisely estimated, with a 95% confidence interval ranging from R1.53 million to R1.56 million, while average annual losses range between R52,920 and R94,302. The relatively narrow intervals suggest low estimation uncertainty and reinforce the economic significance of the phenomenon under study [43].

Descriptive statistics reveal considerable variability in annual cases, with a coefficient of variation of 65.9%, indicating a high dispersion around the mean as indicated in Table 2. This high variability suggests that, despite the overall declining trend, outcomes remain strongly influenced by episodic shocks or discrete events. The coexistence of declining averages and high variability is characteristic of risk-prone systems where exposure is reduced over time, but vulnerability has not been fully eliminated [43,44].

Overall, the results indicate a structural improvement over time, reflected in the declining incidence and reduced frequency of severe outcomes, while persistent variability underscores the need for continued investment in resilience and preventive capacity.

## 5. Discussion

This study provides one of the first long-term economic assessments of malignant catarrhal fever in a South African wildlife–livestock interface context, translating epidemiological evidence into quantifiable direct and indirect economic losses at the farm level. The results demonstrate that malignant catarrhal fever imposes substantial and highly uneven economic costs on cattle producers in Lephalale Municipality, with losses dominated by mortality and strongly shaped by spatial, seasonal, and temporal dynamics. These findings confirm that even low-incidence but highly lethal diseases can generate significant economic burdens, particularly in extensive production systems with limited disease control options.

The predominance of mortality-related losses, accounting for over ninety percent of total economic losses, is consistent with the biological characteristics of malignant catarrhal fever and with findings from pastoral systems elsewhere in Africa. Where mortality represents 81.5% of total losses, treatment accounts for 12.0%, productivity accounts for 2.7%, and milk accounts for 3.8%. Within the direct loss subtotal, mortality contributes 87.1% and treatment 12.9%. The direct losses collectively constitute 93.5% of the total, and the indirect losses constitute 6.5% [5]. Similarly, it has been reported that sudden cattle deaths constituted the principal livelihood impact of malignant catarrhal fever among pastoralists in East Africa, overwhelming other loss components such as treatment or productivity decline. The near irreversibility of mortality losses implies prolonged recovery periods, as herd rebuilding requires time, capital, and access to replacement stock. In communal and smallholder systems where cattle serve multiple economic and social functions, these losses extend beyond immediate income effects to undermine household resilience and asset accumulation, reinforcing a vulnerability to future shocks.

The treatment costs and productivity losses were comparatively modest in magnitude, reflecting both the rapid disease progression and the absence of effective curative options. While supportive treatment may be attempted, its limited efficacy in preventing death constrains the economic returns to treatment expenditure, a pattern also observed in other high-fatality livestock diseases such as heartwater and Rift Valley fever [13,15]. Nevertheless, the persistence of productivity and milk losses, even at lower levels, highlights that surviving animals experience measurable reductions in output that cumulatively affect farm income. These indirect losses are particularly important for small-scale producers who rely on regular production flows for household consumption and cash needs, as noted by [2] in their assessment of livestock sustainability under disease and climate stress.

The strong spatial concentration of cases and deaths in specific wards underscores the role of localized exposure risk that is associated with proximity to wildlife conservation areas and mixed land use patterns. This clustering aligns with historical and contemporary evidence that wildlife reservoirs and seasonal animal movements intensify disease transmission at interface zones [3,4]. The observed spatial heterogeneity suggests that malignant catarrhal fever risk is not evenly distributed across the municipality, supporting the need for geographically targeted surveillance and intervention strategies rather than uniform control measures. Similar spatial concentration has been documented for other interface-associated diseases, including lumpy skin disease and Rift Valley fever, where localized ecological conditions and management practices drive differential risk [14].

Seasonal peaks in the spring and winter further reinforce the ecological underpinnings of disease transmission. These patterns are consistent with known wildebeest calving periods and associated viral shedding, as well as seasonal changes in grazing behavior and animal aggregation. Comparable seasonal dynamics have been reported for malignant catarrhal fever in both Southern and Eastern Africa, where environmental conditions and wildlife behavior jointly shape exposure risk [6,7]. The seasonal alignment between the peak disease burden and the peak economic losses highlights opportunities for anticipatory management, such as heightened surveillance and temporary grazing adjustments during high-risk periods, even in the absence of vaccines.

The long-term decline in reported cases and economic losses after 2010 suggests gradual improvements in disease awareness, surveillance, and management, although these gains appear fragile given the episodic spikes that were observed in earlier years. This pattern mirrors the broader trends in livestock disease control, where sustained institutional investment and knowledge dissemination reduce average disease burden but do not fully eliminate outbreak risk, particularly in systems exposed to ecological variability and land use change [1,28]. The persistence of high variability alongside declining averages indicates that malignant catarrhal fever remains a stochastic shock capable of generating severe losses when exposure conditions align.

From a policy perspective, the findings emphasize the economic rationale for prioritizing preventive and risk reduction strategies over reactive responses. Given that mortality losses dominate total costs, interventions that reduce exposure even marginally may yield substantial economic benefits. However, the feasibility of physical separation between cattle and wildlife remains limited in communal and extensive systems, where fencing and alternative grazing options are constrained. This reinforces the need for integrated land use planning and wildlife–livestock coexistence strategies that explicitly account for animal health externalities, as advocated within the One Health framework [10].

The absence of an effective vaccine for malignant catarrhal fever continues to represent a critical gap in disease control, particularly when contrasted with the demonstrated economic benefits of vaccination for other high-impact livestock diseases such as lumpy skin disease and Rift Valley fever [14]. Until such tools become available, economic evidence such as that generated in this study is essential for informing compensation mechanisms, surveillance prioritization, and farmer support programs in high-risk areas.

This study also offers several methodological contributions relative to previous MCF research in Southern Africa. First, the integration of 21 years of laboratory-confirmed diagnostic surveillance data from the ARC-OVR archive represents an unusually long and continuous dataset, allowing for a temporal, spatial, and seasonal characterization of MCF burden that surpasses the short-term or outbreak-specific datasets typically used in earlier studies. Second, the study applies a component-based economic loss framework that is grounded in animal-health economics, enabling a disaggregated measurement of mortality, treatment, productivity, and milk-related losses. This approach provides a transparent quantification of how different loss pathways contribute to the total economic burden, which has rarely been done for MCF in South Africa. Third, the study combines epidemiological trends and spatial ward-level analysis within a single unified framework, linking case distribution to economic outcomes—an analytical integration not commonly present in the previous MCF literature. Finally, by expressing all monetary values in constant real terms, performing internal consistency checks, and standardizing loss estimates across a long retrospective period, the study enhances comparability and analytical robustness. Collectively, these features distinguish the study methodologically and provide a replicable template for long-term disease-impact assessments in wildlife–livestock interface settings.

## 6. Conclusions

This study quantified the long-term economic burden of malignant catarrhal fever on cattle production systems in Lephalale Municipality over a twenty-one-year period, translating epidemiological outcomes into direct and indirect economic losses. The findings demonstrate that malignant catarrhal fever imposes substantial economic costs despite a relatively low incidence, primarily due to its exceptionally high fatality rate. Mortality losses overwhelmingly dominate the total economic burden, indicating that the disease functions as a severe asset-destroying shock rather than a temporary productivity constraint. These losses are spatially and seasonally concentrated, reflecting the role of wildlife–livestock interfaces, particularly the proximity to wildebeest populations, in shaping disease exposure and outcomes.

The observed decline in cases and economic losses over time suggests gradual improvements in disease awareness and management, yet the persistence of episodic spikes highlights the continued vulnerability of cattle producers in interface areas. For smallholders and communal farmers, the loss of even a small number of animals has far-reaching implications for income stability, herd rebuilding, and household resilience. In the absence of effective vaccines or curative treatments, malignant catarrhal fever remains a structural constraint on livestock development in regions where wildlife conservation and cattle production coexist.

Based on these findings, three policy-relevant recommendations emerge. First, disease surveillance and early warning systems should be spatially targeted toward high-risk wards and intensified during peak seasonal risk periods to enable timely risk communication and preventive action. Second, land use planning and wildlife–livestock coexistence strategies should explicitly incorporate animal health considerations, including the economic costs borne by cattle producers, to support balanced and equitable decision-making in interface zones. Third, sustained investment in the research and development of effective vaccines or alternative control technologies is essential, as even modest reductions in disease incidence would generate substantial economic returns given the dominance of mortality losses.

### 6.1. Limitations of the Study

Despite generating valuable insights into the long-term economic burden of MCF in Lephalale Municipality, the study is subject to several limitations that should be acknowledged.

First, the analysis relied on historical epidemiological and production records whose completeness and accuracy may have varied over the twenty-one-year period. Underreporting of cases, particularly among smallholders and communal farmers, may have resulted in conservative estimates of actual disease incidence and associated economic losses. Second, the economic model focused primarily on quantifiable direct and indirect losses. It did not fully incorporate broader socio-economic spillover effects, such as disruptions to the draught power, asset-based social obligations, transaction costs associated with information-seeking, or psychological impacts on the producers. These unquantified dimensions may contribute meaningfully to the total economic burden of MCF. Third, spatial analysis was constrained by the resolution of available data on wildebeest populations, farm boundaries, and land-use patterns, which limited the precision with which wildlife–livestock interface dynamics could be assessed. Higher resolution ecological and movement data would improve future spatial risk modeling. Fourth, using a constant average cow value over 2001–2021 introduces a measurement error because it does not capture annual price volatility, inflation, or cyclical shocks (e.g., droughts and feed-cost spikes). Consequently, cattle asset values in specific years may be under- or over-estimated, and temporal patterns are smoothed. This limitation affects the level of estimated asset values more than the comparative patterns we analyze.

Lastly, the study did not evaluate the cost-effectiveness of alternative disease management strategies, largely due to the absence of viable vaccines or treatments. This constrains the extrapolation of results into detailed investment decision frameworks.

### 6.2. Recommendations for Future Research

Building on the evidence generated, several avenues for future research are proposed.First, future studies should integrate higher-resolution spatial data, including GPS-based wildlife movement patterns, farm boundary mapping, and seasonal grazing routes, to enhance the understanding of transmission ecology and more precisely identify micro-level risk hotspots.

Second, there is a need for household-level longitudinal studies that capture the socio-economic impacts of MCF beyond the financial losses. Such studies would illuminate how disease-induced livestock mortality affects household resilience, gender roles in livestock management, intergenerational herd transfer dynamics, and long-term livelihood trajectories.

Third, research should explore the feasibility and economic rationale of potential control innovations, including MCF vaccines, targeted vector control (where applicable), and low-cost physical or landscape-level interventions aimed at reducing wildebeest–cattle interactions. Scenario modeling could estimate the expected return on investment of such technologies under varying incidence conditions.

Fourth, further work is needed to assess the governance and institutional mechanisms that mediate disease management at the wildlife–livestock interface, including compensation schemes, land-use zoning, and co-management arrangements between conservation authorities and cattle producers. Fifth, future studies should investigate how climate variability, particularly changes in rainfall patterns, forage availability, and wildlife migration timing, may alter the seasonality and spatial pattern of MCF risk in the coming decades. Furthermore, future work should incorporate year-specific nominal prices deflated to real terms (e.g., CPI/PPI) and, where feasible, province-level series to recover time variation and improve precision. Descriptive statistics and additional robustness checks are reported in Supplementary Table S1.

## Figures and Tables

**Table 1:** The economic loss calculated in detail.

ECONOMIC LOSS CALCULATION DETAILS		
**ECONOMIC PARAMETERS and ASSUMPTIONS**		
**Parameter**	**Value**	**Source/Note**
Case Fatality Rate	85%	Literature: 80–90% average for cattle
Average Cattle Value	R 6698.80	Conservative estimate for African cattle
Treatment Cost per Case	R 837.35	Veterinary consultation and supportive care
Productivity Loss Rate	18%	Body condition loss [5]
Milk Loss Rate	8%	Annual income loss during MCF season
Annual Milk Value per Cow	R 3349.40	Conservative estimate
**LOSS COMPONENTS BREAKDOWN**		
**Component**	**Amount (ZAR)**	**Calculation Method**
**DIRECT LOSSES**		
Mortality Losses	R 1,259,374.00	188 death * 6 698.80
Treatment Costs	R **185,891.70**	222 cases * 837.35
**Subtotal Direct**	**R 1,445,266.10**	**Sum of direct losses**
**INDIRECT LOSSES**		
Productivity Loss	R 40,996.70	34 survivors × 6 698.8 × 18%
Milk Production Loss	R 59,485.30	222 cases × 3 349.4 × 8%
**Subtotal Indirect**	**R 100,482.00**	**Sum of indirect losses**
**TOTAL ECONOMIC LOSS**	**R 1,545,748.10**	**Direct + Indirect**
**SUMMARY STATISTICS**		
**Metric**	**Value**	
Study Duration	21 years	2001–2021
Average Annual Loss	R 73,607.05	Total loss ÷ 21 years
Loss per Positive Case	R 6962.83	Total loss ÷ positive cases
Loss per Death	R 8222.06	Total loss ÷ estimated deaths
Direct Loss Percentage	93.5%	Direct ÷ Total
Indirect Loss Percentage	6.5%	Indirect ÷ Total

**Table 2:** Statistical analysis.

STATISTICAL ANALYSIS RESULTS		
**1. TEMPORAL TREND ANALYSIS**		
**Test**	**Statistic**	**Interpretation**
Pearson Correlation	r = −0.5921, *p* = 0.0047	Significant DECREASING trend over time (*p* < 0.05)
Spearman Correlation	ρ = −0.5913, *p* = 0.0048	Confirms monotonic decreasing trend
**2. PERIOD COMPARISON (2001–2010 vs. 2011–2021)**		
**Metric**	**Value**	
Early Period Mean (2001–2010)	14.40 cases/year	
Late Period Mean (2011–2021)	7.09 cases/year	
T-statistic	2.7733	
*p*-value	0.0121	
**Conclusion**	Significant difference (*p* < 0.05)	Cases decreased significantly in the later period
**3. CONFIDENCE INTERVALS (95%)**		
**Parameter**	**Estimate**	**95% Confidence Interval**
Total Economic Loss	R 1,545,748.00	R 1,532,099.00–R 1,559,397.00
Average Annual Loss	R 73,603.10	R 52,920.50–R 94,302.40
**4. DESCRIPTIVE STATISTICS (Annual Cases)**		
**Statistic**	**Value**	
Mean	10.57	cases/year
Median	11	cases/year
Standard Deviation	6.97	cases
Minimum	1	cases
Maximum	22	cases
Coefficient of Variation	65.9%	High variability

## Data Availability

Data available on request due to privacy/ethical restrictions.

## Supplementary Materials

The following supporting information can be downloaded at: https://www.mdpi.com/article/10.3390/vetsci13030305/s1.
